# Angiotensin Inhibition, TGF-β and EMT in Cancer

**DOI:** 10.3390/cancers12102785

**Published:** 2020-09-28

**Authors:** Fabian Bernhard Pallasch, Udo Schumacher

**Affiliations:** Institute of Anatomy and Experimental Morphology, Center for Experimental Medicine, University Cancer Center, University Medical Center Hamburg-Eppendorf, 20246 Hamburg, Germany; uschumacher@uke.de

**Keywords:** angiotensin inhibition, cancer, cancer-associated fibroblasts (CAFs), desmoplasia, epithelial–mesenchymal transition (EMT), mesenchymal–epithelial transition (MET), stemness

## Abstract

**Simple Summary:**

Angiotensin inhibitors are broadly applied in the treatment of renal and cardiovascular diseases. This review aims to show that these drugs have also been beneficial in cancer therapies. Underlying molecular mechanisms are elucidated. Angiotensin signaling and the antifibrotic properties of inhibiting this signaling are discussed in detail. In essence, these antifibrotic effects are due to crosstalk with TGF-β signaling, which is also described in detail. Due to the altered matrix synthesis by cancer associated fibroblasts under these therapies, TGF-β signaling affects more than just the composition of the extracellular matrix itself, extending to cellular behaviors. Beyond the stroma, TGF-β signaling is also of interest in the epithelial mesenchymal transition, which is also covered.

**Abstract:**

Angiotensin inhibitors are standard drugs in cardiovascular and renal diseases that have antihypertensive and antifibrotic properties. These drugs also exert their antifibrotic effects in cancer by reducing collagen and hyaluronan deposition in the tumor stroma, thus enhancing drug delivery. Angiotensin II signaling interferes with the secretion of the cytokine TGF-β—a known driver of malignancy. TGF-β stimulates matrix production in cancer-associated fibroblasts, and thus drives desmoplasia. The effect of TGF-β on cancer cells itself is stage-dependent and changes during malignant progression from inhibitory to stimulatory. The intracellular signaling for the TGF-β family can be divided into an SMAD-dependent canonical pathway and an SMAD-independent noncanonical pathway. These capabilities have made TGF-β an interesting target for numerous drug developments. TGF-β is also an inducer of epithelial–mesenchymal transition (EMT). EMT is a highly complex spatiotemporal-limited process controlled by a plethora of factors. EMT is a hallmark of metastatic cancer, and with its reversal, an important step in the metastatic cascade is characterized by a loss of epithelial characteristics and/or the gain of mesenchymal traits.

## 1. Introduction

In 1971, US president Richard M. Nixon declared a “war on cancer”, aiming to cure cancer within the next 25 years. Despite huge scientific efforts, the target of finding a cure for cancer was not achieved, as elegantly laid out by the landmark publication of Bailar and Gornik 25 years later [1]. Despite that well-defined molecular targets have been identified, personalized cancer therapies have not been found for all cancer types. Excellent results for one type of therapy were shown in the treatment of Philadelphia chromosome-positive (t(9;22)(q34;q11.2)) chronic myelogenous leukemia (CML) with imatinib as a designed drug that attacks the BCR–ABL fusion product [2,3]. One of the reasons for this success was that this fusion protein is unique to the cancer cells and does not occur in normal cells. In addition, imatinib performs well because CML is a “liquid” cancer without significant intercellular junctions, extracellular matrix (ECM) and without forming cell aggregates, so the drug can easily reach each malignant cell. In contrast to “liquid” cancers, “solid” cancers generate obstacles for drug delivery. It is still un clear which effects are responsible for poor drug penetration into solid tumors [4]. It is likely that the drug penetration problem is a multifactorial one and that the elevated intra-tumoral fluid (IFP) pressure is caused, among other factors, by the molecular composition of the tumor microenvironment itself, abnormal structure of blood and lymphatic vessels and a change in the porosity of the tumor cells caused by alterations of the cell-to-cell contact [4,5]. However, as tumors are very heterogeneous, these factors likely do not all apply to the same extent for all cancer types. For each histological cancer entity, these factors may be of varying importance. In a few cancer entities such as pancreatic ductal adenocarcinomas or subtypes of breast cancer, this barrier seems to be so strong that cancer therapy is failing in nearly every case [6,7]. In In this paper, angiotensin inhibitors are revisited in the context of these alterations in the tumor microenvironment. Angiotensin inhibition is a standard therapy in hypertension, with additional antifibrotic effects being observed [8]. Signaling and effects of different inhibitors, angiotensins, angiotensin receptors and angiotensin-converting-enzymes (ACE) are the focus of this review. The crosslink to transforming growth factor-β (TGF-β) signaling and its impacts on cancer therapy are also covered. The underlying role of TGF-β and the canonical and noncanonical signaling and its general role in cancer biology are elucidated [9]. Furthermore, TGF-β is also a potent inducer of the highly complex epithelial–mesenchymal-transition (EMT), which are also covered.

## 2. Angiotensin Inhibition: How an Old Drug Shows New Tricks

Drugs for inhibition of the renin–angiotensin–aldosterone systems (RAASi) (see Table 1) are standard medication in the therapy of arterial hypertension [10], heart failure [11] and kidney disease, especially those involving kidney fibrosis (e.g., diabetic nephropathy, hypertensive nephropathy) [12,13]. Under physiological conditions, the renin–angiotensin–aldosterone systems (RAAS) maintains the arterial blood pressure, the serum sodium concentration and the extracellular volume [14]. The first step of the RAAS is the release of renin from the juxtaglomerular cell in the kidney. Renin cleaves angiotensinogen to the decapeptide angiotensin I. This decapeptide is then cleaved by the angiotensin-converting enzyme (ACE) to angiotensin II, which acts as a main effector on the angiotensin 1 and 2 receptors (AT1/2), with the AT1 receptor being the key receptor for the vasoconstrictive effects. Further, angiotensin II stimulates the adrenal zona glomerulosa to release the mineralocorticoid aldosterone, which increases renal sodium reabsorption. The RAAS acts via a reduction of the vasoconstrictive angiotensin II signaling, by either inhibiting the ACE (drugs with the suffix -pril), thus the conversion of angiotensin I (AngI) to angiotensin II (AngII) or by blocking the angiotensin I receptor (AT1) (drugs with the suffix -sartan) [14,15].

In conventional therapy, regimes of the above mentioned diseases, the benefits are not solely due to the reduction of blood pressure as angiotensin inhibitors have shown additional cardioprotective and nephroprotective effects by inhibition of collagen synthesis [16,17]. Collagen synthesis is—beyond the cardiovascular and renal system—also of interest during the formation of the cancer stroma. The aim of numerous studies has been to investigate the antifibrotic effect on production of tumor extracellular matrix (ECM) and the following effects in cancer therapy, especially in context of enhancing the drug delivery. Delivery of substances into tissues depends on two basic physical mechanisms, namely diffusion and convection. Convection speed mainly depends on flow from vessels through the tissue into lymphatics. Diffusion speed mainly depends on the molecular size and concentration gradient and is effective over very short distances [18]. Transport within tissues is either dominated by one mechanism or is approximately in equilibrium. Most small molecules like oxygen are mainly transported through diffusion and reach distances of around 100 µm around blood vessels [18,19]. Conversely, the transport of large molecules depends mostly on convection and tissue permeability [20]. A common feature of human tumors is an ECM-rich microenvironment with high density of collagen and hyaluronan. This mass often surrounds nests of tumor cells and increases the distance from blood vessels to tumor cells, thus decreasing drug delivery of even small molecules [21,22]. The growth of tumor cells and matrix lead to solid stress and a consecutive compression of micro vessels, especially lymphatic vessels, leading to decreased convection and increased interstitial fluid pressure. Fluid pressure is also increased by the dense matrix, which increases the flow resistance, leading to impaired convection. Additionally, the dense matrix displays an obstacle for larger molecules, especially nanobodies [5,18,20].

In general, it is well known that RAASi such as losartan reduce the stromal collagen I and hyaluronan synthesis [23]; this reduction is caused by depletion of TGF-β1 signaling [8,24]. In addition to collagen synthesis, TGF-β1 also controls hyaluronan synthesis. It has been shown that hyaluronan synthases 1–3 are decreased in cancer-associated fibroblasts (CAF) after losartan therapy. This observation shows that losartan is at least a trifunctional drug that decreases hypertension, collagen and hyaluronan syntheses. Similar effects have been demonstrated for the ACE inhibitor lisinopril, albeit in comparison with the angiotensin receptor blocker (ARB) losartan, with an inferior collagen I and hyaluronan reduction [23].

The underlying molecular mechanisms for these different actions are well-investigated. Three different angiotensin receptor groups have been found: AT1-, AT2- and Mas-receptor. These receptors belong to the G-protein-coupled receptor family. Angiotensin II is the ligand of AT1 and AT2 receptors. Both receptors stimulate different intracellular pathways and have different, partly opposite effects. The above-mentioned inferiority of lisinopril compared to losartan has been traced back to these opposite AT1 and AT2 effects. This finding was confirmed using AT1 and AT2 receptor knockout mouse models, which were transplanted with the human breast cancer cell line E0771. Compared with wild-type mice, AT1 knockout mice showed less collagen I and hyaluronan deposition in the ECM, and the AT2 knockout mice showed more collagen I and hyaluronan deposition [23,25].

Angiotensin II is cleaved from angiotensin I by the angiotensin-converting enzyme I (ACE I). ACE I is not specific for the latter reaction; it also cleaves bradykinin, substance P, N-acetyl–seryl–aspartyl–lysyl–proline (tetrapeptide AcSDKP) and the luteinizing hormone-releasing hormone [26,27]. Additionally, angiotensin-converting enzyme inhibitors (ACEi) inhibit the production of Ang 1–7. This variety of interactions make ACEi a multifunctional drug with effects well above classical ARBs. The exact effect of ACE on malignant tissues is—due to numerous interactions—nearly unpredictable. This broad spectrum of activities may offer a chance of discovering new drugs by evaluating the property of every substrate and every product of the ACE I, i.e., AcSDKP and substance P. AcSDKP stimulates the secretion of matrix metalloproteinase-1 and is able to induce angiogenesis. Antifibrotic and anti-inflammatory properties have been found in diverse tissues such as heart, lung, liver and kidney [28,29,30,31]. Initially, AcSDKP was described to be a highly potent inhibitor of hematopoietic pluripotent stem cell proliferation [32]. AcSDKP is the cleavage product of thymosin β4 by prolyl oligopeptidase (POP). An increased concentration of AcSDKP has been found to be present in intra-tumoral blood compared to concentrations in normal tissue in human breast, colon head and neck, kidney, lung, skin, ovary and prostate cancer [33]. Additionally, increased AcSDKP plasma levels have been detected in some patients with CLL and AML [34]. Another example is the tachykinin substance P with its G protein-coupled receptor neurokinin-1 receptor (NK1R). Neurokinins have pleiotropic properties (mediators of inflammations, wound healing, leukocyte trafficking, microvasculature permeability and cell survival [35,36,37]), and despite its name it is not only synthesized by neurons, but also by other cell types, such as immune cells (i.e., macrophages/monocytes, dendritic cells, mast cells, neutrophils, natural killer cells, T-lymphocytes) and endothelial cells [38,39,40]. The NK1R antagonist aprepitant shows crosstalk to Wnt signaling in colon cancer cell lines. After administration of aprepitant the Wnt-associated proteins cyclin D1, c-Myc and LEF-1 were downregulated, causing G2 arrest and apoptosis [41]. Additionally, in hepatoblastoma cell lines aprepitant impaired the interaction between Forkhead Box M1 protein with β-catenin, consequently inhibiting Wnt signaling [42]. In breast cancer, substance P application induces matrix metalloproteinase 2 and 14 by enhancing ERK1/2, JNK and AKT pathways, causing invasion and proliferation [37].

**Figure 1:**
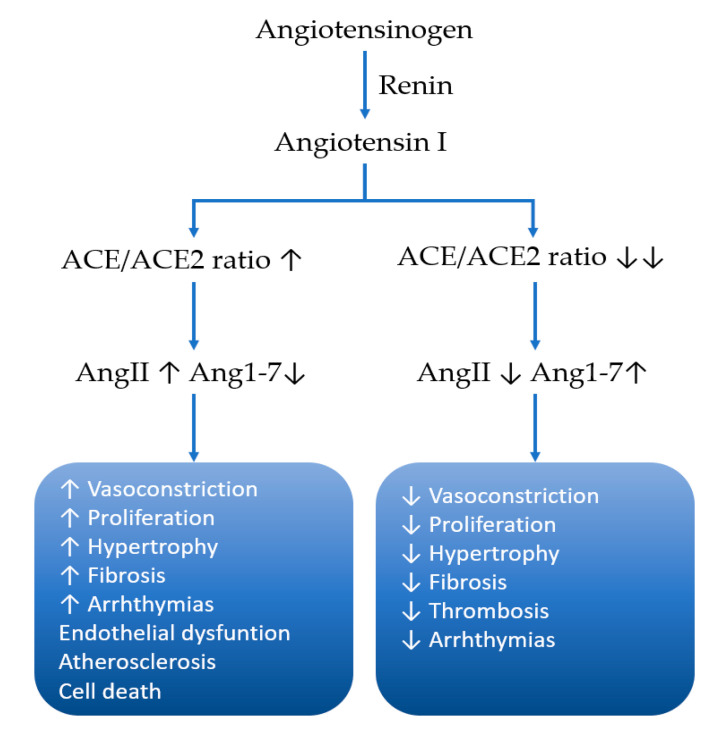
Homeostasis between angiotensin-converting-enzyme (ACE) and angiotensin-converting-enzyme 2 (ACE2) and their products (esp. AngII and Ang(1–7)) determines their net effect on proliferation and blood pressure; adapted from [43].

**Figure 2:**
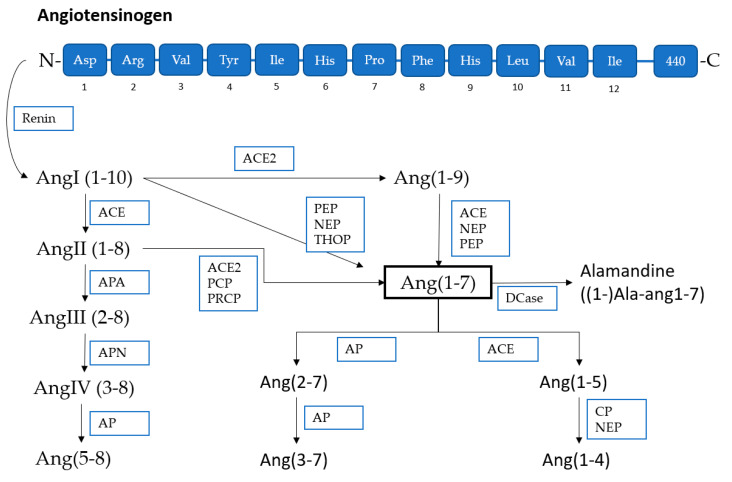
Overview of the cleavage products of angiotensinogen; ACE1/2 (angiotensin-converting enzyme 1/2, aminopeptidase (AP), aminopeptidase A(-N) (APA(-N)) prolyl endopeptidase (PEP), prolylcarboxyendopeptidase (PRCP/PCP), neutral endopeptidase (NEP), carboxypeptidase (CP), decarboxylase (DCase), thimet oligopeptidase (THOP); modified after [45].

The Mas receptor binds angiotensin 1–7 and shows opposite effects of the classical effects of ACE-angiotensin II/AT1. Ang 1–7 is produced by the angiotensin-converting enzyme II, with Ang II as a major substrate. Under physiological circumstances the Mas/Ang 1–7 pathway can be seen as a regulator of the AT1/angiotensin II pathway [43]. Therefore, it has anti-fibrotic, anti-proliferative and anti-hypertrophic properties. In mouse models high dose Ang 1–7 was capable of antagonizing the Ang II effect on blood pressure [44]. In reality, the ACE2/Ang1–7/Mas axis has broader actions, but the complexity of the whole renin–angiotensin–aldosterone system (RAAS) homeostasis and its actions is very impressive and exceeds the aim of this review (Figure 1 and Figure 2). Figure 1 shows the angiotensin homeostasis dependent on the balance between ACE and ACE2 and their consequential effect on the AngII and Ang1–7 equilibrium, thus emphasizing the effect of a shift in this balance, considering fibrosis arrhythmias, vasoconstriction, proliferation hypertrophy, etc. Figure 2 summarizes the cleavage pathways for angiotensinogen, underlining the complexity with multiple involved enzymes and numerous intermediate products.

The hyaluronan-induced vessel compression depends, among other factors, on the collagen content of the tissue. An inverse correlation has been shown between hyaluronan content and perfusion only in collagen rich tumors [23]. This idea was supported by a study by Nieskoski in 2017 when it was proposed that collagen traps the hyaluronan between its fibrils and therefore increases the total tissue pressure [46]. It is proposed that hyaluronan acts like a spring under tension within the collagen trap. This model suggests that the depletion of both ECM components offers a potential target in the improvement of cancer therapy by lowering the total tissue pressure and thereby probably decreasing the interstitial fluid pressure. The benefit of this approach would be expected to correlate with the pressure values and the grade of desmoplasia in ECM [23].

RAASis have a further additional dimensions beyond the interference with production of matrix components; they also seem to decrease the connective tissue growth factor 2 (CCN2). This factor plays a stabilizing role in the generation of ECM fibrosis. This stabilization implies a potential destabilization of the ECM structure by CCN2 inhibition through RAASi, and it yields another additional, yet unevaluated impact for RAASi usage as a potential supporting agent in cancer therapy [23].

Losartan has been found to reduce collagen I production from cancer associated fibroblasts (CAFs) in a dose-dependent manner. In addition, it reduces not only matrix production by CAFs in general, but it also reduces the total amount of CAFs. The molecular background is interference with downstream TGF-β1 signaling by inhibiting thrombospondin-1 synthesis (TSP-1). Angiotensin II induces TSP-1 synthesis via p38 MAPK and JNK signaling and TSP-1 acts as a major activator of TGF-β. Consequently, reduced angiotensin II signaling leads to reduced TSP-1 levels and therefore to reduced TGF-β signaling [47]. This reduction causes reduced solid stress and maybe vessel compression, thus enhancing oxygen and drug delivery [8,23].

The reduction of collagen and hyaluronan content and thereby the potential impaired ECM organization, leads to decreased mechanical forces that contribute to the solid stress within the tumor tissue. Therefore, the latter reduction lowers the compression of blood vessels, improving the delivery of drugs and oxygen. This effect was shown for breast and pancreatic cancer [23]; however, the exact mechanism and the importance of the ECM composition on vessel compression remains unclear. Studies on hepatocellular carcinomas indicate that the vessel decompression results in a clinical benefit due to angiotensin inhibition. A retrospective data analysis revealed a significant improvement in overall survival for patients with hepatocellular carcinoma (HCC) and good hepatic function: Child Pugh class A-treated between 1992 and 2013 with the cancer-specific drug sorafenib, together with inhibitors of the renin–angiotensin system as modulators of the ECM [48]. Similar beneficial effects have been found in mostly retrospective studies on other tumor entities, i.e., breast cancer [49], rectal cancer [50], renal cell carcinoma [51,52], pancreatic cancer [53], lung cancer [54,55] and glioblastoma [56].

There is also evidence for a vascular endothelial growth factor (VEGF)-independent activation of angiogenesis via angiotensin II (Ang II). This evidence led to the hypothesis that angiotensin inhibition could reduce angiogenesis in tumors. Despite known interactions with proangiogenic signaling, the clinical importance of this mechanism remains unclear [23,57].

However, RAASis alone do not affect the median survival in experiments, but in combination with chemotherapy they shown an increased overall survival benefit [8,23,24,48,49,50,51,52,53,54,55,56]. The RAAS and their downstream signaling present an interesting target for new chemotherapeutic drugs augmenting antifibrotic drugs, which could also offer advances for other therapies, such as heart failure and kidney disease.

## 3. TGF-β

**Figure 3:**
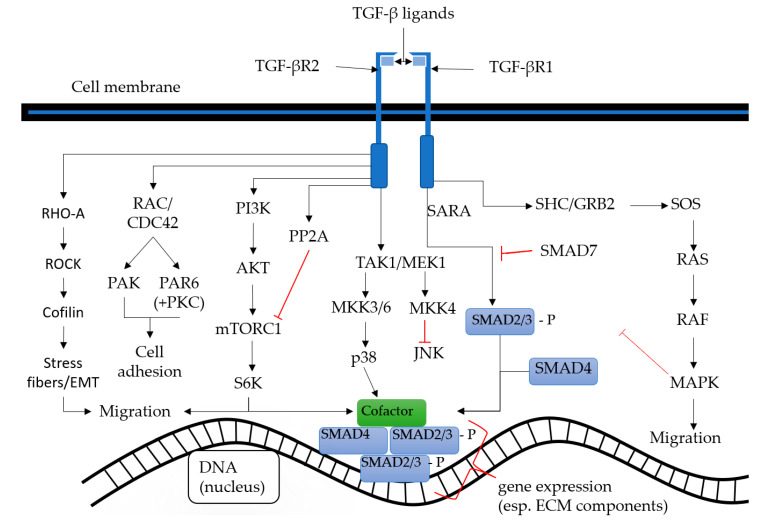
Transforming growth factor-β signaling in a nutshell. Canonical SMAD pathway and the noncanonical crosstalk to other signaling pathways. Partly modified after [86].

As mentioned above, TGF-β1 signaling is reduced by RAASi. TGF-β-signaling is a promising target for drugs because it has an extensive signaling pathway and yields numerous interactions with cancer cells, CAFs and immune response cells. The magnitude of the signaling and possible interactions is shown in Figure 3. TGF-β takes part in different cellular signaling pathways and shows extensive crosstalk in a cellular background-dependent manner. These pathways include signals for important cancer hallmark features such as migration, apoptosis, proliferation and differentiation, including in particular EMT and cancer progression. Collaboration of the WNT and TGF-β pathways are necessary for the induction of EMT, as without WNT signaling TGF-β signaling would cause cell cycle arrest only [58].

The TGF-β signal pathway is engaged in extensive crosstalk with other cell signaling pathways. Its signaling is divided into the canonical SMAD pathway and the noncanonical SMAD-independent pathway. Overall, there are more than 30 members of the TGF-β superfamily, containing bone morphogenetic protein (BMPs), nodal, activin and inhibin [9,59].

The canonical pathway is executed in cancer cells, as well as in associated stromal cells, the cancer-associated fibroblasts (CAF). CAFs are a main player in the formation of the tumor microenvironment and are associated with increased growth, metastatic potential and chemotherapy resistance. TGF-β1 is frequently secreted by tumor cells and is in general the main player within tumor cell-stroma cell crosstalk. Once CAFs are induced they produce TGF-β1 themselves, leading to an auto-/paracrine stimulus for both cancer and stroma cells, maintaining fibrogenesis [60,61].

In the canonical TGF-β signal pathway the TGF-β ligand binds to the receptor Type 2; its binding to the receptor recruits and activates the receptor Type 1. This receptor, in turn, phosphorylates the C-terminal SXS-motif of receptor-regulated SMAD2/3 (r-SMAD). This phosphorylation is now required for the formation of a heterotrimeric complex, which is necessary for efficient nuclear accumulation and the initiation of sufficient signaling through interactions with common-mediator SMAD4 (co-SMAD) and SMAD-binding elements (SBE) in target gene promoters (5′-GTCT-3′-4bp SMAD box) [62,63,64,65,66]. Most common is the signaling through SMAD3/4 complexes, while SMAD 2 plays a less important role in SMAD signaling. The affinity between SMADs and the SBEs is very low, therefore additional transcription factors and transcriptional co-activators and co-repressors are recruited to form a multi-transcription factor complex, facilitating a stable interaction with the DNA. The SMAD 4-associated transcription factors are Runx-related proteins, FoxH1, Mixer and E2F; associated co-activators are CBP and P300 and associated Sloan Kettering Institute (SKI) co-repressors and Ski novel (SnoN) [67,68]. Furthermore, the SMAD3 binding needs nucleosome-depleted areas for DNA binding. Altogether, SMAD3 binds in a cell-type specific manner at different DNA sequences, mostly mediated through master transcription factors (i.e., OCT4, Myod1, PU.1) to promote a cell-specific TGF-β response [65,69,70]. SMAD6 and SMAD7 belong to the group of inhibitory SMADs (I-SMAD) which prevent phosphorylation of r-SMADs. SMAD7 signaling is considered to be a negative feedback loop from SMAD 2/4, 3/4 complexes [66,71,72]. The signaling is summarized in Figure 3.

The effect of TGF-β on cancer cells themselves still remains elusive. A change of TGF-ß action during different stages of malignant progression is proposed. In normal epithelial cells TGF-β has growth-inhibitory and apoptosis-promoting properties, this role persisting during early tumor stages. In many tumor entities cancer cells lose the negative response on TGF-β, and they develop antagonistic properties such as a pro-oncogenic and pro-metastatic ones [73]. Alterations in the TGF-β signaling cascade have been found in various cancer entities. The term “alterations” includes point mutations, but also gains, amplifications, deletions and DNA methylation. Forty-three genes of the canonical TGFβ superfamily and 50 SMAD downstream targets from the “PanCancer cohort” were analyzed with 33 entities from 9125 patients. Within the 43 canonical TGFβ genes 39% displayed an alteration in at least one gene. An alteration frequency of over 50% was found in 12 tumor types, cutaneous melanoma had with 70% the highest frequency, while the lowest was found with 4% in thyroid carcinoma. Their mRNA analysis of the 50 downstream genes revealed that in all mutations the direction of changes in target genes was the same. This finding implies that every change was directed, either in increasing or decreasing activity, across a heterogenous population of tumor types [74].

Due to its extensive complexity, there are numerous possible alterations and co-alterations, and they have been shown to occur at different levels of the signaling cascade. Starting at the ligand level, they include TGF-ß amplification, transforming growth factor receptor 2 (TGFBR2) mutations at the receptor level, SMAD4 mutations at the signal transduction level, and finally, further on downstream, SMAD4 target gene level. As seen in gliomas, the homozygote deletion of the cyclin-dependent kinase inhibitor 2 (CDKN2B), a tumor-suppressive target of the SMAD pathway, was found to be mutated [9,74,75,76,77].

The tumor stroma consists of connective tissue, which is in its composition, especially in desmoplastic tumors, not unlike scar tissue [78]. In this non-malignant tissue TGF-β acts in its physiological key role in initiating wound healing, scar formation and induction of fibrosis. Especially in wound healing it was shown that TGF-β signaling is a key inducer of fibrosis. Remarkedly, it is released within five minutes after epithelial damage. As mentioned above, cancer cells can act like these injured epithelia and release TGF-β. The SMAD3 pathway is the key pathway for TGF-β response in fibroblasts. TGF-β release stimulates production of further profibrotic molecules in fibroblasts, neutrophils and macrophages [79,80,81,82,83]. TGF-β is an inducer of CCN2 (a.k.a., connective tissue growth factor (CTGF), which takes part in important TGF-β effects, such as fibroblast proliferation and production and stabilization of ECM [23,82].

Compared to epithelial cells, TGF-β has opposing effects on stromal mesenchymal cells. TGF-β stimulates cell proliferation and ECM secretion of fibroblasts/myofibroblasts. TGF-β promotes the transition from fibroblasts to contractile myofibroblasts. These myofibroblasts are designated as cancer-associated fibroblasts in malignant tissues [84]. Numerous matrix and membrane proteins are upregulated through TGF-β, i.e., collagen I–V, laminin entactin, perlecan, elastin, fibronectin, hyaluronan and osteopontin. Additionally, TGF-β has negative effects on the degradation of matrix by decreasing the activity of matrix metalloproteases (MMPs), which are matrix-degrading enzymes [85,86,87].

The effect of TGF-β on the tumor microenvironment can be summarized as induction of fibrosis by enhancing ECM structural proteins, ECM-modulating enzymes and depleting ECM-degrading enzymes. These alterations lead to increased matrix stiffness and physical forces within the tissue, causing solid stress, blood and lymphatic vessel compression and altered gene expression in cancer cells and other stromal cells [88,89]. The compressive stress reaction of cancer cells includes enhancement of invasive and metastatic properties, resulting in an increase in malignancy [86]. These alterations in the tumor microenvironment have important implications for therapy and therapy resistance. The desmoplastic character of some cancer entities with cell nests surrounded by a dense collagen rich stroma leads to increased diffusion distance and a steric barrier, presumably especially in the use of macromolecular drugs. There are a few therapeutic strategies targeting TGF-β signaling, such as neutralizing antibodies, ligand traps, antisense-oligonucleotides, kinase inhibitors and immune strategies [90]. As TGF-β was shown to be a potent inducer of EMT and is crucial for maintaining EMT, its role during EMT is described below.

## 4. EMT

Carcinomas develop from epithelial cells, which are characterized by structural intercellular junctions, namely adherens junctions, tight junctions and gap junctions. This feature of intercellular junctions in epithelial tissues can be considered as antimigratory, anti-invasive and therefore antimetastatic. In order to become metastatic, it is functionally necessary to lose these intercellular junctions in order to acquire a migratory phenotype. Loss of epithelial characteristics causes consecutively enhanced motility, loss of apical–basal polarity, detachment from the basement membrane, and in general a partial or a complete mesenchymal phenotype. During this process, which is called EMT, the cells simultaneously display mesenchymal and epithelial traits. Cancer cells thereby recapitulate, at least in part, classical EMT, which is a physiological process during embryogenesis, especially during gastrulation. The term “classical” thereby implies a complete linear transformation from an epithelial cell to a mesenchymal one at the end of the process. Cancer cells, and in particular metastatic ones, can use this conserved embryonic differentiation program. EMT in cancer cells is often incomplete; however, the loss of epithelial characteristics is large enough for the definition of EMT, even without a complete switch to classical mesenchymal characteristics. In order to distinguish between the different types of EMT, the term “partial EMT” was coined [91]. One of the first experimental observations of EMT in adult non-malignant cells was observed in a gastrulation-independent EMT during lens development, with lens epithelial cells adapting mesenchymal traits in a collagen matrix [92].

EMT was considered to be a binary state of cells, especially during gastrulation, for a long time. However, recent findings have shown that epithelial–mesenchymal hybrid states exist that are not exclusively found in cancer cells, as this process can also take place during organogenesis and wound healing. Due to the linear appearance in gastrulation, the initial term coined was epithelial–mesenchymal transformation, which was accordingly changed to transition to accommodate the existence of partial EMT states [93].

The old binary view on EMT grounds on an oversimplification in experimental design, especially with considerations of the EMT marker expression, which does not reflect the occurrence of intermediate stages during this process (e.g., most commonly used markers: epithelial: E-cadherin, occludins and cytokeratins; mesenchymal: vimentin and N-cadherin) [94]. The co-expression of mesenchymal und epithelial markers in one cell led to the hypothesis that the process in not necessarily binary and also yields a first definition of partial EMT states. To define partial EMT in one cell only by its co-expression of epithelial and mesenchymal markers would be an oversimplification, because paradoxically for partial EMT the expression of mesenchymal markers is not a necessity [94]. For partial EMT, the loss of epithelial hallmarks (e.g., apical–basal polarity, E-cadherin) is sufficient and seems to appear especially in early tumor stages [95]. Further, classical epithelial markers may remain initially untouched. EMT typically starts with altering cell polarity and thereby affects tight junctions, adherens junctions, desmosomes and gap junctions [95,96]. In cancer cells the extent of EMT is very heterogenous and also varies within different tumor entities [94,97]. The loco typico of intensified EMT processes within a malignant tumor is found in the invasion front. At this front it is difficult to separate a mesenchymal cancer cell that has undergone EMT from an original tumor stroma cell, which is naturally of mesenchymal descent. This differentiation problem was solved by cell lineage labeling. This approach provided experimental in vivo evidence of spontaneous EMT. A Cre/LOX system was used, with a FSP1-Cre (fibroblast specific protein), WAP-Cre (whey acidic protein) and an upstream stop cassette floxed LacZ reporter, which is histochemically visualized by blue X-Gal. It was crossed into three different tumor models (WAP-Myc, MMTV-PyMT, MMTV-neu). The stroma surrounding the tumor in the WAP-Myc model stained blue after X-gal staining. There were blue stained spindle-shaped cells within the stroma, and further immunostaining revealed that some of these cells express fibronectin or fibronectin and cytokeratin or fibronectin, cytokeratin and E-cadherin [98,99].

E-cadherin is frequently suppressed in epithelial cells undergoing EMT and therefore represents a main hallmark of the transition of epithelial cells into mesenchymal ones during EMT, but other epithelial intercellular junction molecules are suppressed too, such as ZO-1, occludin, claudin-1/-7, plakophilin and desmoplakin [95,100]. These proteins are all junctional proteins and their downregulation results in a loss of attachment, which is necessary for further motility, invasion and thus metastasis formation.

However, despite all this evidence for the vital role of EMT during metastasis formation, there is further evidence that metastasis can develop independently of EMT, for example by using the podoplanin pathway and remodeling of the cytoskeleton [101] or via vessels that encapsulate tumor clusters (VETC) in HCC [102] or via COL1A1 in breast cancer [103]. Despite these studies, the role of EMT-independent metastasis remains controversial, as EMT is widely viewed to be essential in the metastatic cascade [104] and furthermore, has implications for other cancer hallmarks, e.g., stemness and drug resistance [105].

As outlined above, in metastatic deposits re-expression of epithelial marker/epithelial junctions is observed, hence metastases resemble the epithelial phenotype of the primary tumor. This reversal of the EMT is called mesenchymal–epithelial transition (MET), yielding adhesive tumor cells, forming new epithelial differentiated metastases [98]. The necessity of MET in the metastatic cascade has been extensively investigated. The human bladder carcinoma cell line TSU-Pr1 with both mesenchymal and epithelial traits was used to generate a progression series by inoculating the original cell line orthotopically, intracardiac or intratibial into immunodeficient mice. The progression cell lines TSU-Pr1 B1 were obtained from a single bone metastasis from the original TSU-Pr1 after intracardial injection of tumor cells in SCID mice. This was repeated with the TSU-Pr1 B1 cell line, yielding TSU-Pr1 B2. TSU-Pr1 has a fibroblastoid phenotype; with the progression series the cells developed an epithelial character and lost mesenchymal traits (e.g., vimentin expression). The metastases all resembled an epithelial phenotype. Additionally, the orthotopic inoculated mesenchymal cells produced more micrometastases compared to the epithelial phenotype cells and vice versa, the intracardiac or intratibial inoculation, skipping the initial metastatic steps, was favorable for metastases formation from epithelial phenotype cells [104,106].

In pancreatic ductal adenocarcinoma small metastases displayed a more mesenchymal phenotype, whereas with increasing size of metastases, displayed a more epithelial character, convenient to MET [107]. The epithelial glycoprotein-2 was determined within different cell lines (three SCLC, three ovarian, three colon), in cell culture and after transplantation in SCID mice. In the early metastases the glycoprotein was found to be transiently downregulated, indicating a transient loss with probably general reexpression of epithelial markers [108].

### 4.1. Extracellular Factors Regulating EMT

EMT is a highly complex biologic process, there are many factors promoting and inhibiting EMT. The crosstalk between all promotors and inhibitors determines the extent and permanence of the transition. For example, TGF-β 1,2,3 receptor blockade displayed chemostatic effects in this context, simultaneously decreasing the degree of mesenchymal markers as a hallmark of EMT inhibition, underlining the great influence of TGF-ß in tumor biology [100,109]. TGF-β may be the most famous inducer of EMT. There are several other important EMT promoting factors such as hypoxia, Wnt, IL-6, Notch, FGF, EGF, HGF, PFGF, UV light, hydrostatic pressure, tension and shear stress [100,110,111,112]. The latter are all signals acting on an extremely complex equilibrium of factors influencing the degree of transition on a variable spectrum. The following downstream signaling implies an orchestra of factors, including transcription factors, RNA interference, epigenetic modifications, alternative splicing, subcellular localization and protein stability [94].

### 4.2. Transcription Factors Regulating EMT

**Figure 4:**
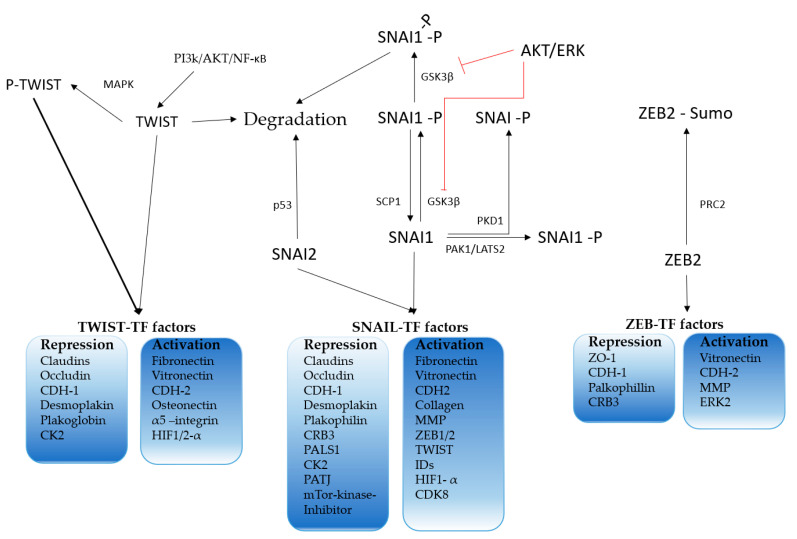
Key regulation of the core epithelial–mesenchymal transition (EMT)-activating-transcription factors (EMT-TFs) and their major targets. Modified from [96].

EMT underlies an extensive influence of environmental factors upon the cancer cells, necessitating control and crosstalk between different cells orchestrated by EMT-activating-transcription factors (EMT-TFs). The EMT-TF core family consists of SNAI1, SNAI2, TWIST1, ZEB1 and ZEB2. EMT-TF are widely seen as the master regulators of EMT [113,114,115] (Figure 4). These TFs were shown to have impact in nearly all cancer stages and are known drivers of malignancy. They can have both inductive and repressive features at the same time, depending on whether they induce an epithelial or mesenchymal phenotype [116]. As they are responsible for the maintenance of the epithelial or mesenchymal phenotype, respectively, they are also expressed to a varying degree, depending on the cell type, in normal tissues [117,118].

The different core EMT-TFs have different functions, and the activation clusters determine the degree of transition and hence the phenotype of the cell. For example, TWIST1 acts on trend as a potent mesenchymal inducer, but has a low epithelial repressing potency. Interestingly, TWIST 1-induced regulation has been shown to directly influence the cellular phenotype of cancer cells at the tumor invasion front. TWIST 1 has been found regulating invadopodia and actin cytoskeleton in invading tumor cells [119]. In contrast, SNAI1 and ZEB1 show a mostly low potency in induction of mesenchymal features, but a high potency for epithelial repression. Thus, SNAI1 and SNAI2 were found to suppress E-cadherin expression in different (cancer) cell lines [120,121]. Furthermore, in SNAI1-overexpressing MDCK cells, an E-cadherin-independent suppression of tight junction proteins was found (e.g., ZO-1, different claudins). This suppression was found both on the transcriptional and on the translational level. SNAI1-overexpressed MDCK cells displayed a mesenchymal phenotype, and ectopic E-cadherin reexpression alone was unable to reverse the phenotype, indicating that SNAI1 was the regulator of the epithelial phenotype and E-cadherin its executor. Additionally, SNAI1 acted on survival pathways (MEK/ERK and PI3K/AKT) and the antiapoptotic BCL-xl expression was increased [122,123,124]. A set of SNAI1 potentially regulated genes is summarized in Figure 4. As mentioned before, the displayed genes are possibly regulated, depending on cell background, yielding an individual reaction set for every tumor type and EMT-TF. For example, SNAI1 or TWIST1 were knocked out in pancreatic cancer cells (KPC), with no effect on invasion or metastasis formation being observed [125]. Nevertheless, an increased gemcitabine sensitivity was found after the knock-out. This led to the hypothesis that EMT is not a necessity for metastasis formation [125]. Another group found in the same mouse model a reduction in the number of metastases through ZEB1 knock-out [126]. This observation emphasizes the impact and effect-heterogenicity of single EMT-TFs.

ZEB1 was found to be a driver of malignancy in melanomas, resulting in poor patient outcome [127]. ZEB2, another core EM-TF from the ZEB family, displayed opposite effects in mouse melanomas; ZEB2 decreased the malignant progression and the loss of ZEB2 increased it [127]. This finding can be explained by the physiological role of ZEB proteins in melanocytes. ZEB1 is important for maintenance of the stem cell character of melanoblasts, whereas ZEB2 acts as a differentiation signal in these cells. The loss of ZEB2 decreased the microphthalmia-associated transcription factor, a key inducer of differentiation in melanocytes [117]. The oncogenic properties of ZEB1 were enhanced by TWIST1, whereas SNAI2 was associated with increased ZEB2 tumor suppression [117,127]. These studies underline the complicated interactions of EMT-TF, not only in the different effects in different cellular background, but also in the co-expression and crosstalk of different EMT-TFs themselves.

TGF-β is an inducer of EMT, especially in the context of wound healing and closure of disrupted epithelial sheets. During regeneration, TGF-β induces via SNAI2 a migratory partial EMT phenotype within epithelial cells, as observed in the migratory front of keratinocytes on the wound border [128,129].

As implied previously, EMT-TF can also take effect on different pro- and antiapoptotic genes, such as the BCL-2 family [130]. In breast cancer and epithelial ovarian cancer cells SNAI1/2 repressed p53 and anti- and proapoptotic p53 effector proteins, resulting in a suppression of the physiological DNA damage response [131,132]. In a cisplatin resistance study in head and neck cancer, SNAI1 was shown to induce the excision repair cross complementation Group 1 protein (ERCC1), resulting in increased resistance [133]. In FaDu hypopharyngeal cancer cells, TWIST1- overexpressing cancer cells showed a decreased sensitivity to taxol, due to an increased p-glycoprotein activity and impaired apoptosis following increased BCL-2 and decreased Bax and cleaved caspase-3 and -9 [134].

**Figure 5:**
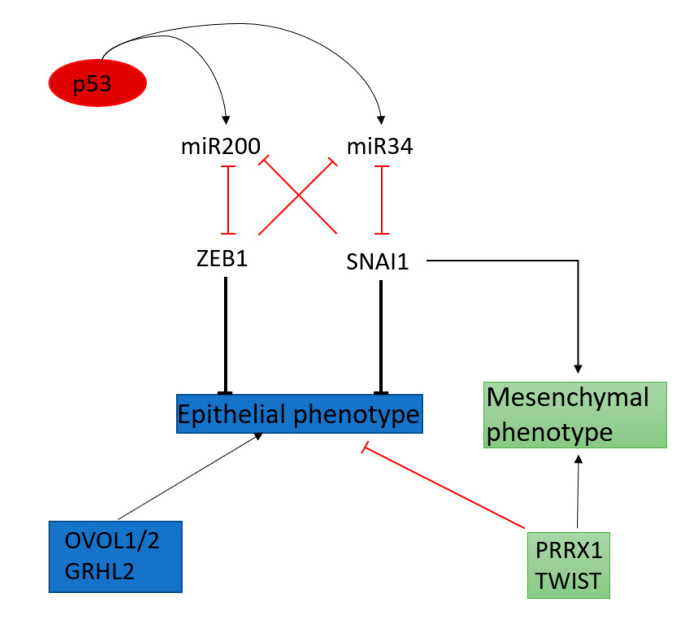
Core of the double-negative feedback loop between EMT-TF and microRNA. Modified from [94].

The regulation of the EMT-TFs does not only involve proteins, but also miRNAs (see Figure 5) which have either negative or double negative (i.e., positive) feedback loops, as shown in Figure 5 [94,135].

In addition to the classical EMT-TF, a growing number of minor EMT-inducing or EMT-enhancing proteins have been uncovered [96]. They seem to directly induce EMT under certain circumstances or have been found to cooperate with core EMT-TFs. For example: Tead2–Yap–Taz, E12-47, SOX4, SOX9, FOXC2, FOXA2, FOXF2, HMGA2, PRRX1, ZNF281 [96,136,137,138,139]. The basal breast cancer cell line FOXC2 was found to delocalize E-cadherin into the cytosol, induce N-cadherin, vimentin, fibronectin, αSMA and to regulate canonical TGF-β signaling [138]. Forced expression of Tead2 in Py2 T murine breast cancer cells decreased the expression of E-cadherin and ZO-1, while increasing the levels of vimentin, ZEB1, ZEB2 and SNAI2 and altering the cytoskeleton from cortical actin-to-actin stress fibers [136].

There are also EMT-inhibiting transcription factors such as grainyhead-like 2 (Grhl2) and Ovol1/2 [94]. Due to the many different transcription factors regulating EMT, it is at present impossible to determine a universal applicable rule for the effect of EMT-TF in cancer cells. The effect and importance of distinct TFs are highly dependent on the cellular background and they vary between stage and tumor type.

### 4.3. EMT and Stemness

The interplay between EMT and stemness are discussed using the intestinal stem cells as a model system. The intestinal stem cells (ISC) reside in a niche within the intestinal crypts. This niche protects cells from TGF-β signaling; as mentioned above TGF-β has cytostatic properties on epithelial cells. The niche also contains mitotic and stem cell-maintaining signaling, such as WNT, NOTCH and EGF. Differentiated Paneth cells residing in an adjacent location at the base of the crypts express the latter factors and are important for the integrity of the stem cell niche, as upon removal of Paneth cells, the stem cells also disappear. If the stem cells are destroyed, the niche itself is able to re-induce stem cells from progenitor cells, as seen in vitro [140,141]. Similar in vivo experiments in other stem cell compartments have shown even further potential in regaining stemness. After destruction of basal cells in the mouse trachea, fully differentiated club cells regained stemness and refilled the stem cell niche [142]. Similar findings were made in renal tubule restoration after damage, where differentiated tubular epithelial cells transiently dedifferentiated and proliferated to repair the damage, after which the cells re-differentiated [143]. Therefore, cancer stem cells (CSCs) are not only a very promising target for cancer therapy, but they are also subject to EMT. The transporter for diphtheria toxin was brought under the control of LGR5—the intestinal stem cell marker—within a colorectal cancer xenograft model. As expected, the toxin killed the stem cell marker-positive cells, consequently leading to decreased tumor growth. LGR5-negative proliferative cells tried continuously to replenish the LGR5+ stem cell pool. After therapy cessation the primary tumor recovered, but the distant metastases did not, indicating that potent stem cell niches are mainly found in the primary tumor [144]. At the invasive border of squamous cell carcinomas, the EMT inducer TGF-β was found to slow down the proliferation of CSCs, resulting in resistance against antiproliferative therapy. Lineage tracking showed that these cells rebuild the tumor after cisplatin treatment [144]. Similar data were obtained from CSC in breast and skin cancer, subpopulations of those cells that had undergone EMT, resulting in more migratory phenotype with slow cell proliferation [145].

EMT is often seen as a necessity for metastasis formation as this differentiation program induces an invasive and migratory phenotype, with an increased metastasis-initiating potential. Once established as disseminated tumor cells in a distant organ, the formation of clinically detectable metastases requires a reverse EMT, namely MET, and thus silencing of EMT-TF occurs, resulting in an epithelial phenotype of the metastasis. Consequently, tumor cells fixed in an epithelial EMT phenotype have been shown to elicit very poor metastatic potential [146,147]. Therefore, interplay between EMT and stemness is important.

One example for direct interplay of EMT and stemness was found in immortalized human mammary epithelial (HMLEs) cells. TWIST1 or SNAI1 were expressed ectopically in these cells. The TWIST1 or SNAI1 induction lead to an CSC-like/mammary stem cell status with an CD44^high/^CD22^low^ phenotype [148]. In mammosphere assays, following EMT, cells displayed more than a 30-fold increase in the number of mammospheres. In a similar study, with transiently-induced TWIST1 in HMLEs, after the signaling was stopped the cells regained an epithelial phenotype, but stem-like properties remained stable, measured by the ability to form mammospheres [149].

Considering in vivo appearance of metastasis, resembling an epithelial phenotype after switching from EMT to MET, EMT can be mainly seen as a context-dependent positive or negative differentiation program for stem cells. This process is supported by the physiological properties of stem cells and the growth inhibition through TGF-β in the ISC niche, as mentioned above. For example, there is experimental evidence that TWIST1 can induce stable stem-like cells in mammary epithelial cells [149]. Nevertheless, in general EMT has a temporo-spatial limitation within the metastatic cascade, so does the inhibition of stem cells. As a result, epithelial phenotypic metastasis can be formed. In general, there is data supporting both the inhibitory and stimulating influence of EMT on stemness or on stem-like properties. These data were obtained in different contexts (inhibitory: [146,150] stimulating: [96,151]).

Beyond the extensive transcriptional regulation, there is an overlying network of influences, such as epigenetic (mentioned above; histone modification, DNA methylation) posttranscriptional (alternative splicing, miR-sponge, processing), translational (stability polyA-tail, initiation) and posttranslational (ubiquitylation, acetylation, phosphorylation), which all also influence EMT/MET transitions.

## 5. Conclusions

Cancer remains a common cause of death, and thus it may be helpful to revisit some old standard medications under new aspects, in order to repurpose them to improve cancer treatment. Angiotensin inhibitors are such candidate drugs—especially those acting on AT-1 signaling, which has been found to be an important pro-fibrotic signaling. The angiotensin receptor blockage is able to reduce TGF-β1 signaling, therefore decreasing collagen I and hyaluronan synthesis, resulting in reduced desmoplasia and probably increased drug delivery [23]. The effect of a direct receptor blockade on desmoplasia is greater than the effect of blockade of the converting enzyme, most likely due to the different AT-receptors and plethora of proteins cleaved by ACE-I. The analyses of retrospective clinical studies from different entities indicate that these effects are not theoretical only but are clinically apparent in an increased overall patient survival. TGF-β is pivotal in the communication of cancer cells with cancer-associated fibroblasts and with the induction and maintenance of the latter, TGF-β builds a positive auto-stimulatory feedback loop within CAFs. There are a considerable number of potential new drugs in development targeting TGF-β, aiming from different angles (antibodies, ligand traps, antisense-oligonucleotides, kinase inhibitors, immune strategies) [90]. TGF-β is also a potent inducer of EMT. EMT is characterized through loss of epithelial characteristics and/or gain of mesenchymal characteristics and together with its counterpart MET, they are broadly known as key players in metastasis formation. Especially in cancer, the transition lies within a limited spatiotemporal spectrum between epithelial and mesenchymal cells. The degree of transition is regulated by numerous mechanisms, such as extracellular signals, transcription factors, microRNAs, alternative splicing and epigenetics. The plethora of factors contributing to EMT offer several potential new drug targets. However, later steps of the metastatic cascade impede the usage of anti-EMT drugs, as these drugs could even facilitate the formation of metastasis, because the reversal of EMT the MET is seen in metastasis. Therefore, EMT-inhibitors could block the early steps within the metastatic cascade, while promoting the later steps of the cascade [94]. In conclusion, further research is needed in order to win the war on cancer; hence we may need to further re-evaluate older drugs within the context of cancer.

## Figures and Tables

**Table 1:** List of important angiotensin inhibitors.

ACE Inhibitor (-pril)	AT1 Blocker (-sartan)
Ramipril	Candesartan
Enalapril	Losartan
Captopril	Olmesartan
Lisinopril	Telmisartan
Perindopril	Valsartan
	Irbesartan

Angiotensin-converting enzyme (ACE); Angiotensin-receptor-1 (AT-1), for details see text.
